# Supplementary material for: Platelet-Rich Plasma in Alopecia Areata—A Steroid-Free Treatment Modality: A Systematic Review and Meta-Analysis of Randomized Clinical Trials
Source: Biomedicines. 2022 Jul 29;10(8):1829. doi: 10.3390/biomedicines10081829 (PMC9405156; doi:10.3390/biomedicines10081829)
Supplement: Supplementary file 1 [file biomedicines-10-01829-s001.zip › biomedicines-1753776-supplementary.pdf]

Supplementary Online Content

F.A. Meznerics, K. Illés, F. Dembrovszky, P. Fehérvári, L.V. Kemény, K.D. Kovács, N.M. Wikonkál, D. Csupor, P. Hegyi, A. Bánvölgyi. **Platelet-rich plasma in alopecia areata – a steroid-free treatment modality. A systematic review and meta-analysis of randomized clinical trials. Archives of Dermatological Research**

Corresponding author: András Bánvölgyi MD, PhD
Department of Dermatology, Venereology and Dermatooncology, Faculty of Medicine, Semmelweis University, Budapest, Hungary
Postal address: 41 Mária utca, Budapest, H-1085, Hungary
E-mail address: banvolgyi.andras@med.semmelweis-univ.hu
Telephone number: +36 1 266-0465

Supplementary Results: Secondary outcomes

Figure S1: Risk of bias assessment of SALT score outcomes of the studies included in meta-analysis, using the revised tool for assessing risk of bias in randomized trials (Rob 2)

Figure S2: Risk of bias assessment of SALT score outcomes of the studies included in meta-analysis, broken down to tools, shown in percentage

Figure S3: Risk of bias assessment of SALT score outcomes of the studies included in systematic review, using the revised tool for assessing risk of bias in randomized trials (Rob 2)

Figure S4: Risk of bias assessment of SALT score outcomes of the studies included in systematic review, broken down to tools, shown in percentage

Figure S5: Risk of bias assessment of adverse effects outcomes of the studies included in systematic review, using the revised tool for assessing risk of bias in randomized trials (Rob 2)

Figure S6: Risk of bias assessment of adverse effects outcomes of the studies included in systematic review, broken down to tools, shown in percentage

Figure S7: Risk of bias assessment of administration-related pain outcomes of the studies included in systematic review, using the revised tool for assessing risk of bias in randomized trials (Rob 2)

Figure S8: Risk of bias assessment of administration-related pain outcomes of the studies included in systematic review, broken down to tools, shown in percentage

Figure S9: Risk of bias assessment of recurrence rate outcomes of the studies included in systematic review, using the revised tool for assessing risk of bias in randomized trials (Rob 2)

Figure S10: Risk of bias assessment of recurrence rate outcomes of the studies included in systematic review, broken down to tools, shown in percentage

Figure S11: Risk of bias assessment of dermoscopic evaluation outcomes of the studies included in systematic review, using the revised tool for assessing risk of bias in randomized trials (Rob 2)

Figure S12: Risk of bias assessment of dermoscopic evaluation outcomes of the studies included in systematic review, broken down to tools, shown in percentage

Figure S13: Risk of bias assessment of Ki-67 level outcomes of the studies included in systematic review, using the revised tool for assessing risk of bias in randomized trials (Rob 2)

Figure S14: Risk of bias assessment of Ki-67 level outcomes of the studies included in systematic review, broken down to tools, shown in percentage

Figure S15: Risk of bias assessment of burning/itching sensation outcomes of the studies included in systematic review, using the revised tool for assessing risk of bias in randomized trials (Rob 2)

Figure S16: Risk of bias assessment of burning/itching sensation outcomes of the studies included in systematic review, broken down to tools, shown in percentage

Supplementary References

Supplementary Results: Secondary outcomes

Dermoscopic evaluation

Hegde *et al*. found a decreasing trend in dermoscopic grading in platelet-rich plasma (PRP), triamcinolone acetonide (TrA) and placebo groups compared to baseline, though it was not statistically significant. There was no significant difference between PRP and TrA group, nor PRP and placebo group [1].

Trink *et al*. reported a decrease of dystrophic hairs in both PRP and TrA groups. Comparing PRP to TrA, PRP led to significantly better dermoscopy results ($p<0.001$) [2].

Fawzy *et al*. showed significant improvement in trichoscopic findings comparing the baseline and post-treatment parameters in both PRP and TrA groups regarding the number of follicular units per opening ($p = 0.027$, $p = 0.007$), black dots ($p = 0.007$, $p = 0.003$), broken hairs ($p = 0.046$, $p = 0.008$) and dystrophic changes ($p = 0.003$, $p = 0.014$). Exclamation marks and tapered hairs showed a significant improvement in PRP group ($p = 0.008$), but not in TrA group. The detection of teleangiectasiae was significantly higher in TrA group ($p = 0.007$) [3].

Ki-67 levels

Both PRP and TrA significantly increased the levels of Ki-67 in alopecia areata (AA) patches compared to placebo, and Ki-67 levels were significantly higher after PRP treatment compared to TrA ($p<0.05$) [2].

Burning/itching sensation related to AA

Trink *et al*. reported that both PRP and TrA decreased the itching/burning sensation of the patients enrolled ($p<0.001$) [2].

Figure S1: Risk of bias assessment of SALT score outcomes of the studies included in meta-analysis [1, 3-5], using the revised tool for assessing risk of bias in randomized trials (Rob 2)

Study	Experimental	Comparator	Outcome	Weight	Randomization process	Deviations from intended interventions	Missing outcome data	Measurement of the outcome	Selection of the reported result	Overall
Albalat, 2019 [4]	PRP	TrA	SALT score	1	?	+	+	+	+	!
Fawzy, 2020 [3]	PRP	TrA	SALT score	1	?	+	+	+	+	!
Hegde, 2020 [1]	PRP	TrA	SALT score	1	+	+	+	?	+	!
Kapoor, 2020 [5]	PRP	TrA	SALT score	1	?	+	+	?	+	!

+ Low risk
? Some concerns
– High risk

PRP-platelet-rich plasma, TrA-triamcinolone acetonide, SALT score-Severity of Alopecia Tool Score

Figure S2: Risk of bias assessment of SALT score outcomes of the studies included in meta-analysis [1, 3-5], broken down to tools, shown in percentage

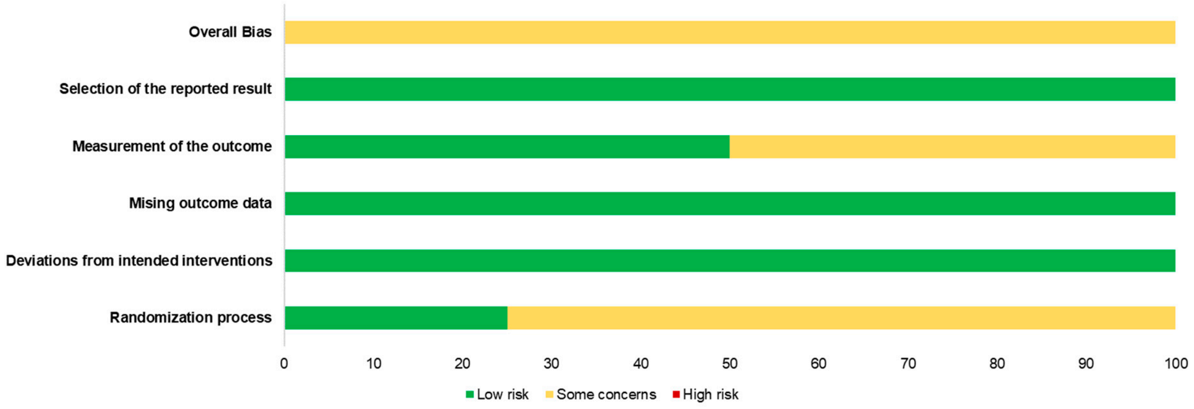

Figure S3: Risk of bias assessment of SALT score outcomes of the studies included in systematic review [1, 2, 6], using the revised tool for assessing risk of bias in randomized trials (Rob 2)

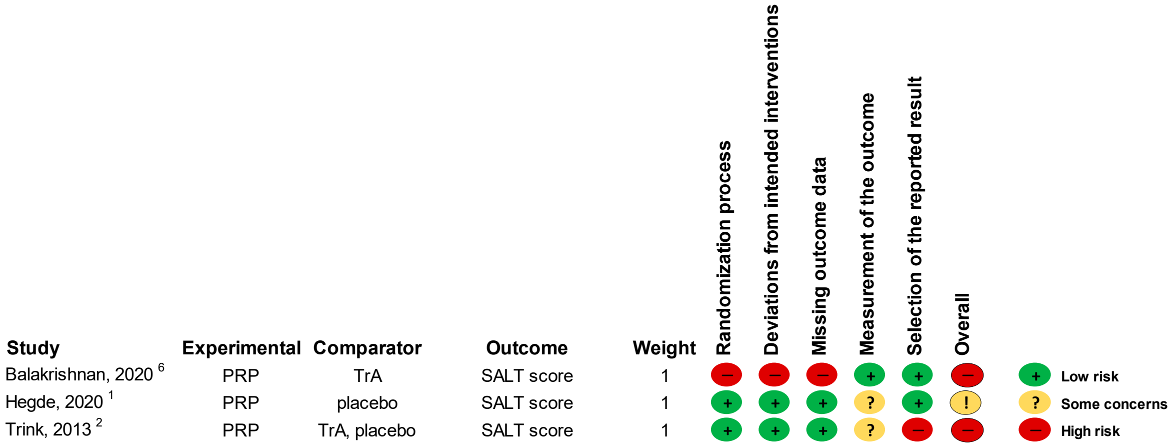

PRP-platelet-rich plasma, TrA-triamcinolone acetonide, SALT score-Severity of Alopecia Tool Score

Figure S4: Risk of bias assessment of SALT score outcomes of the studies included in systematic review, broken down to tools, shown in percentage

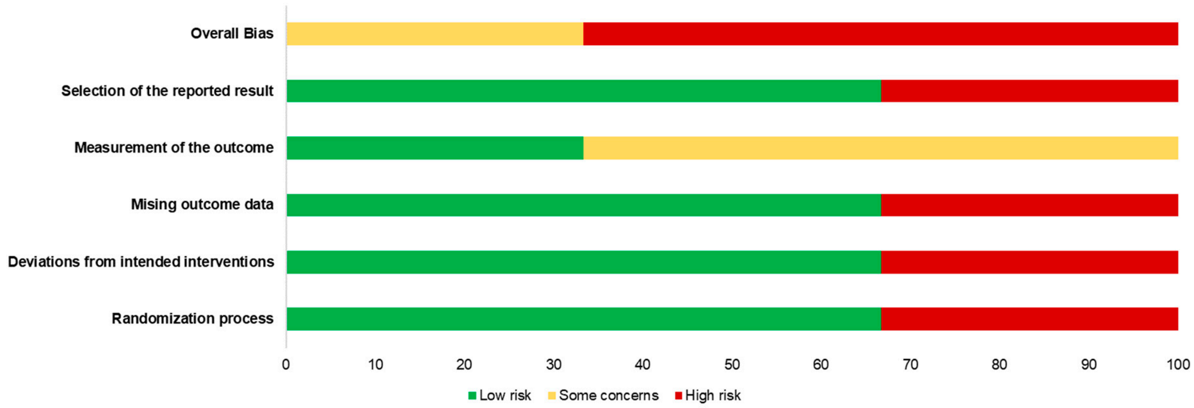

Figure S5: Risk of bias assessment of adverse effects outcomes of the studies included in systematic review [1, 2, 4-6], using the revised tool for assessing risk of bias in randomized trials (Rob 2)

Study	Experimental	Comparator	Outcome	Weight	Randomization process	Deviations from intended interventions	Missing outcome data	Measurement of the outcome	Selection of the reported result	Overall
Albalat, 2019 [4]	PRP	TrA	Adverse effects	1	?	+	+	+	+	!
Balakrishnan, 2020 [6]	PRP	TrA	Adverse effects	1	−	−	−	+	+	−
Hegde, 2020 [1]	PRP	TrA, placebo	Adverse effects	1	+	+	+	+	+	+
Kapoor, 2020 [5]	PRP	TrA	Adverse effects	1	?	+	+	+	+	!
Trink, 2013 [2]	PRP	TrA, placebo	Adverse effects	1	+	+	+	+	+	+

+ Low risk
? Some concerns
− High risk

PRP-platelet-rich plasma, TrA-triamcinolone acetonide

Figure S6: Risk of bias assessment of adverse effects outcomes of the studies included in systematic review [1, 2, 4-6], broken down to tools, shown in percentage

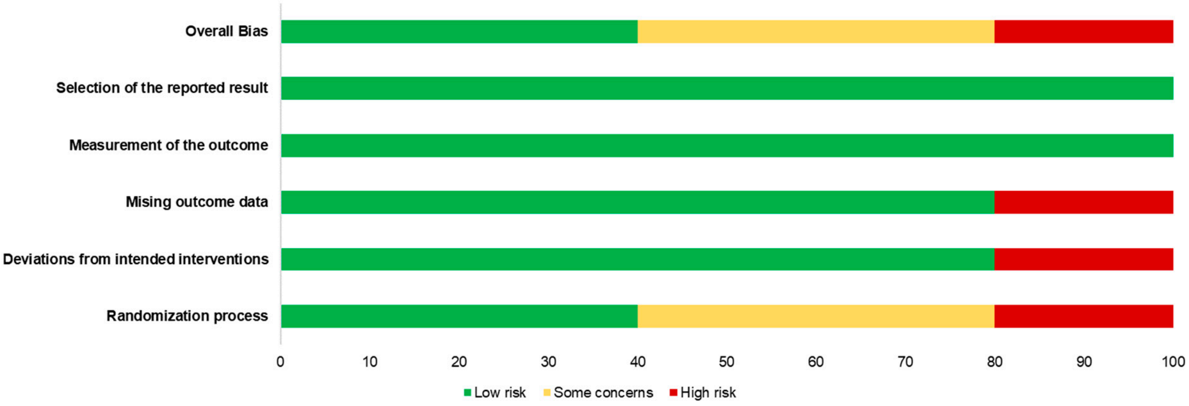

Figure S7: Risk of bias assessment of administration-related pain outcomes of the studies included in systematic review [1, 5, 6], using the revised tool for assessing risk of bias in randomized trials (Rob 2)

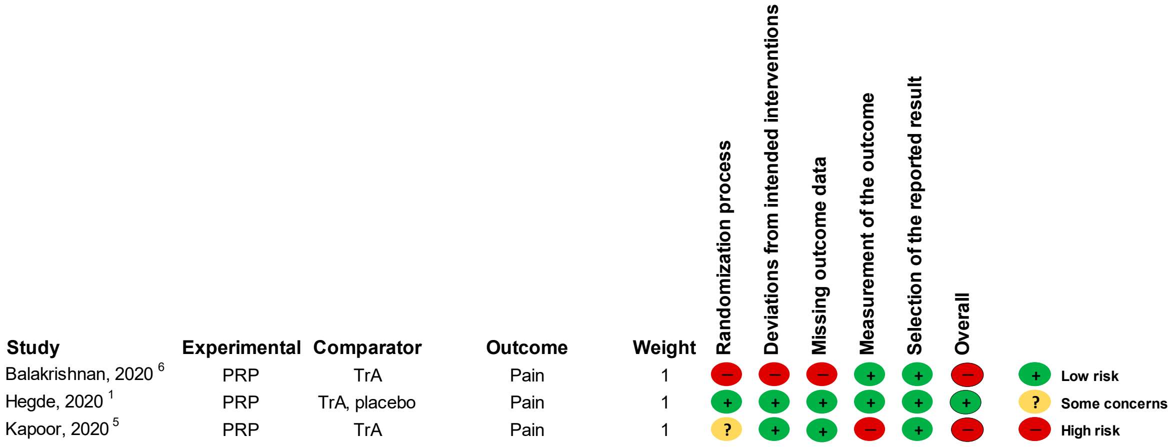

PRP-platelet-rich plasma, TrA-triamcinolone acetonide

Figure S8: Risk of bias assessment of administration-related pain outcomes of the studies included in systematic review [1, 5, 6], broken down to tools, shown in percentage

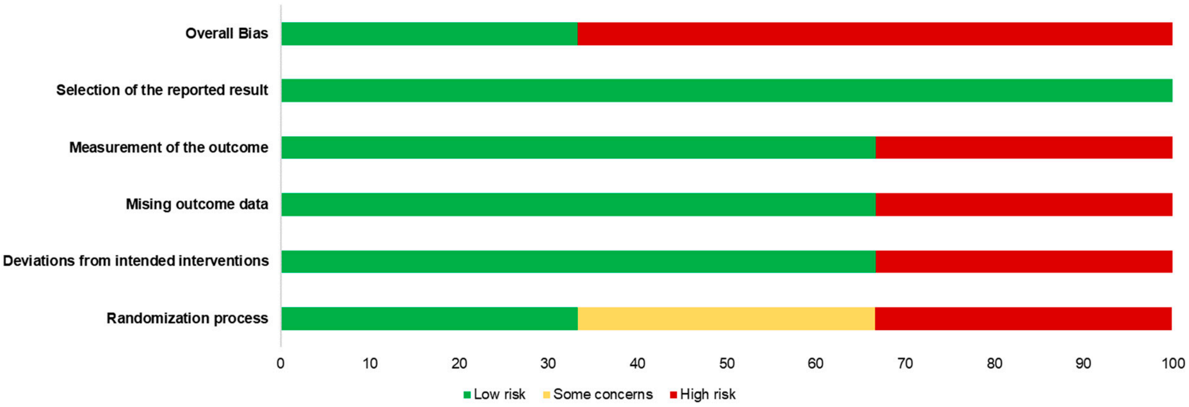

Figure S9: Risk of bias assessment of recurrence rate outcomes of the studies included in systematic review [2, 4], using the revised tool for assessing risk of bias in randomized trials (Rob 2)

Study	Experimental	Comparator	Outcome	Weight	Randomization process	Deviations from intended interventions	Missing outcome data	Measurement of the outcome	Selection of the reported result	Overall
Albalat, 2019 [4]	PRP	TrA	Recurrence rate	1	?	+	+	+	+	!
Trink, 2013 [2]	PRP	TrA, placebo	Recurrence rate	1	+	+	+	+	+	+

+ Low risk
? Some concerns
– High risk

PRP-platelet-rich plasma, TrA-triamcinolone acetonide

Figure S10: Risk of bias assessment of recurrence rate outcomes of the studies included in systematic review [2, 4], broken down to tools, shown in percentage

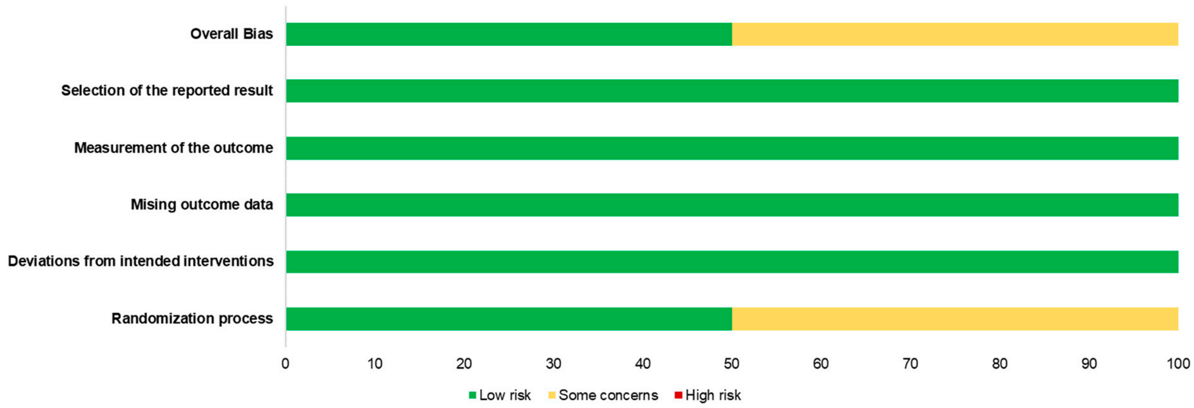

Figure S11: Risk of bias assessment of dermoscopic evaluation outcomes of the studies included in systematic review [1-3], using the revised tool for assessing risk of bias in randomized trials (Rob 2)

Study	Experimental	Comparator	Outcome	Weight	Randomization process	Deviations from intended interventions	Missing outcome data	Measurement of the outcome	Selection of the reported result	Overall
Hegde, 2020 [1]	PRP	TrA, placebo	Dermoscopic evaluation	1	+	+	+	?	+	!
Trink, 2013 [2]	PRP	TrA, placebo	Dermoscopic evaluation	1	+	+	+	?	+	!
Fawzy, 2020 [3]	PRP	TrA	Dermoscopic evaluation	1	+	+	+	+	+	+

+ Low risk
? Some concerns
− High risk

PRP-platelet-rich plasma, TrA-triamcinolone acetonide

Figure S12: Risk of bias assessment of dermoscopic evaluation outcomes of the studies included in systematic review [1-3], broken down to tools, shown in percentage

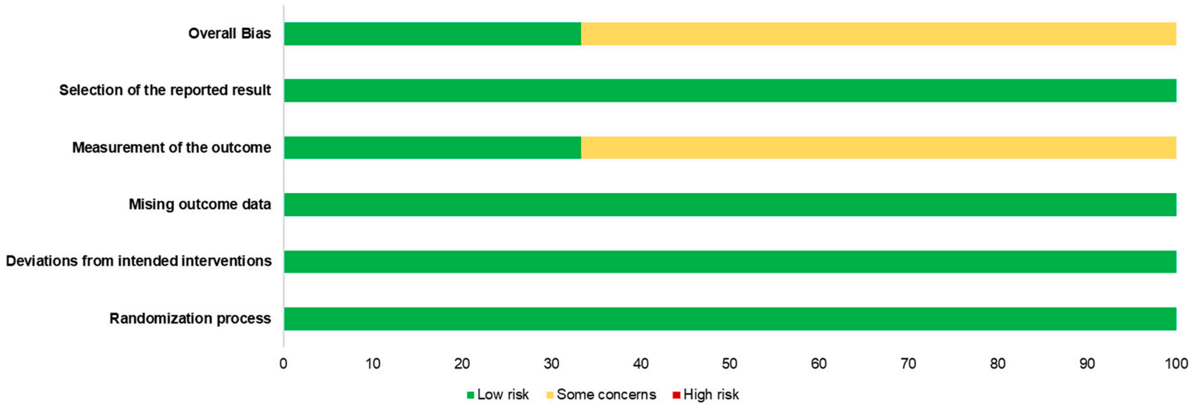

Figure S13: Risk of bias assessment of Ki-67 level outcomes of the studies included in systematic review [2], using the revised tool for assessing risk of bias in randomized trials (Rob 2)

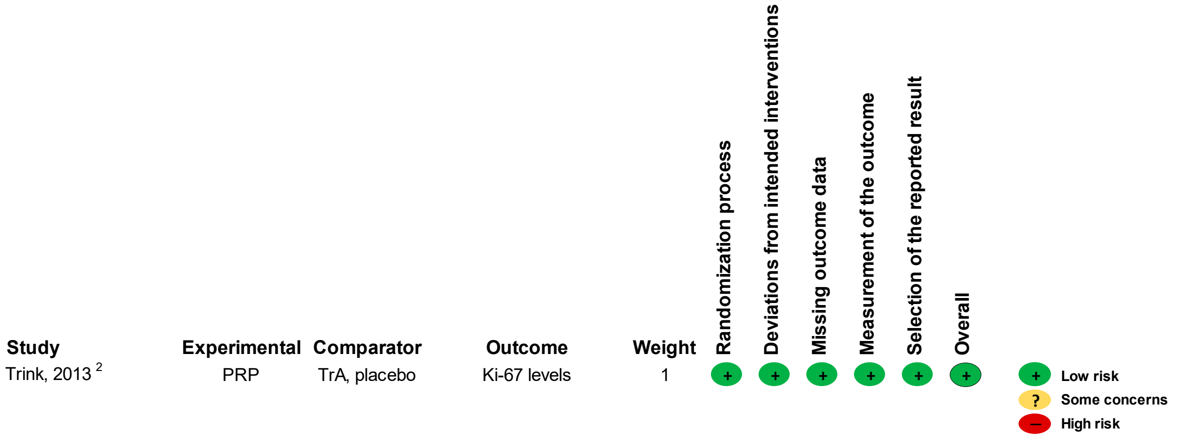

PRP-platelet-rich plasma, TrA-triamcinolone acetonide

Figure S14: Risk of bias assessment of Ki-67 level outcomes of the studies included in systematic review [2], broken down to tools, shown in percentage

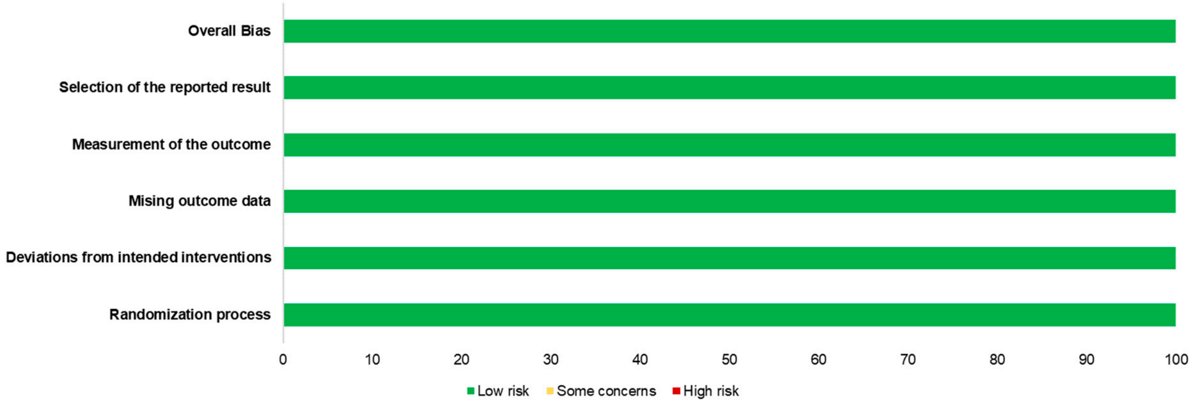

Figure S15: Risk of bias assessment of burning/itching sensation outcomes of the studies included in systematic review [2], using the revised tool for assessing risk of bias in randomized trials (Rob 2)

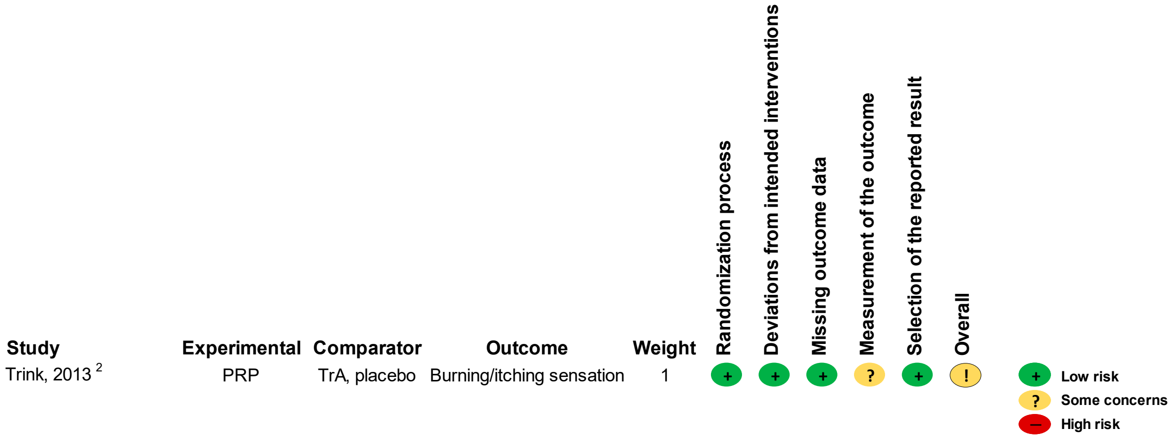

PRP-platelet-rich plasma, TrA-triamcinolone acetonide

Figure S16: Risk of bias assessment of burning/itching sensation outcomes of the studies included in systematic review [2], broken down to tools, shown in percentage

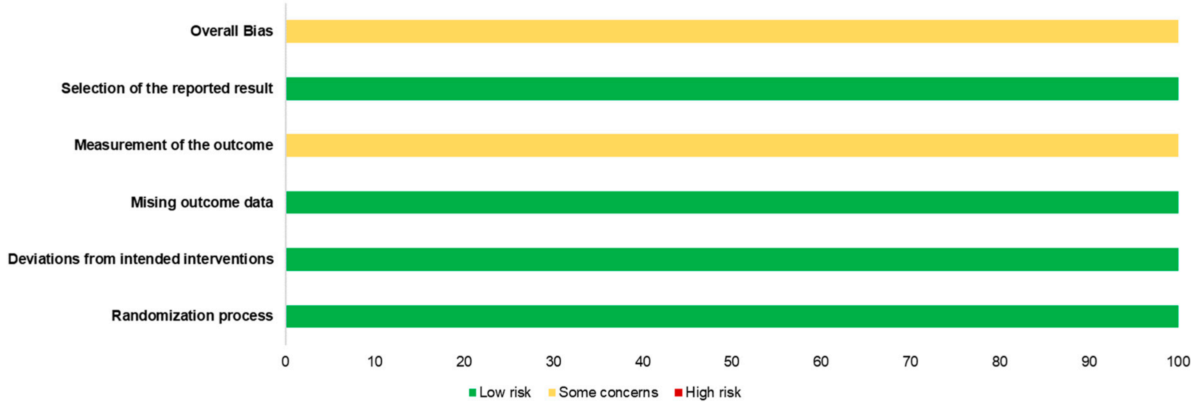

Supplementary References

1. Hegde, P., V. Relhan, B. Sahoo and V. K. Garg. A randomized, placebo and active controlled, split scalp study to evaluate the efficacy of platelet-rich plasma in patchy alopecia areata of the scalp. *Dermatologic therapy* **2020** *33*: e14388.
2. Trink, A., E. Sorbellini, P. Bezzola, L. Rodella, R. Rezzani, Y. Ramot and F. Rinaldi. A randomized, double-blind, placebo- and active-controlled, half-head study to evaluate the effects of platelet-rich plasma on alopecia areata. *British journal of dermatology* **2013** *169*: 690-94.
3. Fawzy, M. M., R. Abdel Hay, F. N. Mohammed, K. S. Sayed, M. E. D. Ghanem and M. Ezzat. Trichoscopy as an evaluation method for alopecia areata treatment: A comparative study. *Journal of Cosmetic Dermatology* **2021** *20*: 1827-36.
4. Albalat, W. and H. M. Ebrahim. Evaluation of platelet-rich plasma vs intralesional steroid in treatment of alopecia areata. *Journal of cosmetic dermatology* **2019** *18*: 1456-62.
5. Kapoor, P., S. Kumar, B. K. Brar, N. Kukar, H. Arora and S. K. Brar. Comparative evaluation of therapeutic efficacy of intralesional injection of triamcinolone acetonide versus intralesional autologous platelet-rich plasma injection in alopecia areata. *J Cutan Aesthet Surg* **2020** *13*: 103-11.
6. Balakrishnan, A., B. Joy, A. Thyvalappil, P. Mathew, A. Sreenivasan and R. Sridharan. A comparative study of therapeutic response to intralesional injections of platelet-rich plasma versus triamcinolone acetonide in alopecia areata. *INDIAN DERMATOLOGY ONLINE JOURNAL* **2020** *11*: 920-24.
